# Pediatric oncology drug development and dosage optimization

**DOI:** 10.3389/fonc.2023.1235947

**Published:** 2024-01-29

**Authors:** S. Y. Amy Cheung, Justin L. Hay, Yu-Wei Lin, Rik de Greef, Julie Bullock

**Affiliations:** Certara, Princeton, NJ, United States

**Keywords:** pediatric, oncology, dosage optimization, Project Optimus, model informed drug development, clinical trial design, modeling and simulation

## Abstract

Oncology drug discovery and development has always been an area facing many challenges. Phase 1 oncology studies are typically small, open-label, sequential studies enrolling a small sample of adult patients (i.e., 3-6 patients/cohort) in dose escalation. Pediatric evaluations typically lag behind the adult development program. The pediatric starting dose is traditionally referenced on the recommended phase 2 dose in adults with the incorporation of body size scaling. The size of the study is also small and dependent upon the prevalence of the disease in the pediatric population. Similar to adult development, the dose is escalated or de-escalated until reaching the maximum tolerated dose (MTD) that also provides desired biological activities or efficacy. The escalation steps and identification of MTD are often rule-based and do not incorporate all the available information, such as pharmacokinetic (PK), pharmacodynamic (PD), tolerability and efficacy data. Therefore, it is doubtful if the MTD approach is optimal to determine the dosage. Hence, it is important to evaluate whether there is an optimal dosage below the MTD, especially considering the emerging complexity of combination therapies and the long-term tolerability and safety of the treatments. Identification of an optimal dosage is also vital not only for adult patients but for pediatric populations as well. Dosage-finding is much more challenging for pediatric populations due to the limited patient population and differences among the pediatric age range in terms of maturation and ontogeny that could impact PK. Many sponsors defer the pediatric strategy as they are often perplexed by the challenges presented by pediatric oncology drug development (model of action relevancy to pediatric population, budget, timeline and regulatory requirements). This leads to a limited number of approved drugs for pediatric oncology patients. This review article provides the current regulatory landscape, incentives and how they impact pediatric drug discovery and development. We also consider different pediatric cancers and potential clinical trial challenges/opportunities when designing pediatric clinical trials. An outline of how quantitative methods such as pharmacometrics/modelling & simulation can support the dosage-finding and justification is also included. Finally, we provide some reflections that we consider helpful to accelerate pediatric drug discovery and development.

## 1 Introduction, regulatory landscape and motivations

In 2030, the United States is expected to witness approximately 2.3 million new cancer cases, which is around a 45% increase from 1.6 million in 2010, and globally around 29 million cases by 2040, out of which less than 1% will be diagnosed in pediatric patients, as reported by recent statistics (1–3). Nevertheless, pediatric cancer is the second most common cause of death in children aged 1 to 14 in the U.S. after accidents, and its incidence has been steadily increasing (1). Pediatric cancer is a complex and heterogeneous disease with over 100 different types, and its incidence varies by age, with certain types being more prevalent in younger age groups, such as leukemias. In contrast, other conditions, such as lymphomas, are more frequent in adolescents (4). A rare disease is often a result of genetic mutation and has a low prevalence rate (5); e.g., a disease is rare when it affects less than 1 in 2,000, as defined by the European Union (6). Childhood cancer in children and adolescents (over 100 types) is a rare disease with a low number of regulatory-approved therapies or widespread use of innovative therapies (e.g., targeted or immunotherapy) (7). A framework for better dosage finding through advanced clinical trial design and innovative quantitative approaches for pediatric oncology populations, for example, with the application of biomarker-driven trial is needed (8, 9).

Despite hundreds of approved cancer therapies, only ~50 drugs ranging from cytotoxic chemotherapy (CTx) agents to molecularly-targeted therapies (10) have pediatric labelling in over 70 indications. This discrepancy in available therapies for pediatric compared to adult cancers highlights the unmet and urgent need to develop more effective treatments for pediatric cancer patients. This urgent need is also reflected in the recent US legislation Research to Accelerate Cures and Equity (RACE) for Children Act of 2017 (11), which authorizes the Food and Drug Administration (FDA) to require pediatric clinical trials for new oncology drugs that have a mechanism of action (MoA) relevant to the pediatric population. Since then, in June 2022, specific types of combination studies were also added to the legislation of the RACE Act, effectively encouraging the sponsor provide strategic drug development plans to develop mono and combination therapies for the pediatric population (12, 13). Early initiation of pediatric studies also includes information on whether an investigated product is safe and efficacious for the pediatric population and hence provides a go/no-go decision.

Project Optimus was introduced by the FDA’s Oncology Center of Excellence (OCE) to reform the dosage selection and optimization framework in oncology development. More recently, this has been supported by draft guidance issued by the FDA (14). Adult oncology development often employs a rule-based approach to support dose escalation and de-escalation in Phase 1; traditionally, the goal has been identifying the maximum tolerated dose (MTD) to take forward to Phase 2 as the recommended Phase 2 dose (RP2D) (15). The RP2D is likely the dose taken forward in testing in Phase 3 studies. In addition to the overall challenges and limitations of dosage findings in oncology, another unique consideration for oncology is that the patient population in Phase 1 studies are patients, mostly all comers who failed previous treatment and several prior lines of therapies.

The MTD approach paradigm does not allow the optimization and justification of the best dosage to take forward by balancing the risk/benefit ratio as well as one would desire. The information collected during non-clinical studies and key clinical information beyond safety is either considered independently or is not propagated to the next stage of drug development and eventually omitted in the dose justification argument. As for adult oncology drug development, pediatric oncology development follows the rule-based and MTD approach. In addition, the age differences between adults and pediatrics are typically taken care of by a simpler, scaled MTD approach, and the subsequent escalation/de-escalation steps to establish the pediatric MTD would be again rule-based driven. As a result, only one dose and regimen will be evaluated post Phase 1.

The FDA Project Optimus, RACE Act and other regulatory initiatives (16) allow drug developers to create a new paradigm and strategy to tackle pediatric drug development. These new regulatory requirements and legislation also impact how drug developers devise binding regulatory documents such as Pediatric Investigation Plans (PIPs) for Europe and initial Pediatric Study Plans (iPSPs) for the USA. These include the evidence collection through non-clinical models, e.g., identification of appropriate cell lines and animal models, and the application of modeling and simulation/model-informed drug development (MIDD) to evaluate pharmacokinetic (PK)- pharmacodynamic (PD) or exposure/response (ER) relationships (17).

In this review article, we provide some key considerations when designing oncology clinical trials in pediatric populations and development strategies that can utilize all the available information with the support of quantitative approaches using PK-PD/ER to optimize the dosage in oncology pediatric populations. We will also outline the typical challenges, opportunities, and key questions associated with model-informed approaches to support the trial design and dose justification in pediatric oncology drug discovery and development with a few examples from the literature. Finally, we will share some recommendations for the future, which incorporate the vision of FDA Project Optimus that continues to support the RACE Act.

## 2 Regulatory landscape

Pediatric Investigation Plans (PIPs) and initial Pediatric Study Plans (iPSPs) serve as critical regulatory instruments to ensure the appropriate assessment of medicinal products in children. PIPs and iPSPs are comprehensive documents outlining pediatric populations’ development and testing requirements. These plans are essential for generating robust data on the safety and efficacy of drugs in children that will address this population’s specific needs and vulnerabilities. Unless the investigated drug qualifies for an exemption or waiver, PIPs are required in the European Union (EU) for all new medicinal products. iPSPs, on the other hand, are required by the FDA for drugs intended for adult and pediatric populations. Sometimes, waivers or deferrals may be sought for PIPs and iPSPs. Waivers may be requested when it is scientifically justified that certain studies or assessments are unnecessary or unfeasible for a given drug or indication in the pediatric population. Deferrals allow for the postponement of specific studies until after the drug’s initial approval for adult use, provided that specific criteria are met. The granting of waivers or deferrals requires a rigorous evaluation by regulatory authorities, considering factors such as disease prevalence, available treatment options, and ethical considerations.

With regards to adolescents, a review from Leong et al. (18) evaluated a number of approved drugs between 2015 to 2021 and concluded that selection of an appropriate dosage for adolescents should ultimately be determined based on available PK or PD data with consideration of body size effect on drug exposure, toxicity, and efficacy data (if available) and the therapeutic index of the drug, and dose- and exposure-response relationships in adults. These recommendations for adolescents are repeated in the US Food and Drug Administration (FDA) Guidance on Inclusion of Adolescent Patients in Adult Oncology Clinical Trials (19). These guidelines and publications have been successful to encourage developers to include adolescents (> 12 to < 18 years of age) in disease and target-appropriate adult oncology trials (20): “*Of 2764 identified trials, 2176 were included: 79% adult, 19% transitional, 2% pediatric., …, For trials investigating targeted therapies, this increase was 460% (197 trials available at age 17 years; 901 at 18 years) and for immunotherapies, 1200% (55 at age 17 years; 658 at 18 years)”*.

The RACE Act is a recently enacted legislation designed to promote the development of more effective treatments for pediatric cancers. Incorporated as Title V in the 2017 U.S. FDA Reauthorization Act, the RACE Act builds upon earlier advances made by the Pediatric Research Equity Act (PREA) and the Best Pharmaceuticals for Children Act (BPCA), which led to the labeling of more than 800 medicines for pediatric use but had limited success with oncology drugs. Previously, PREA was triggered only by an application for a new indication, dosage form, dosing regimen, route of administration, or active ingredient, unless the drug was for an indication with orphan designation. The RACE for Children Act amends PREA and mandates that the sponsor of an original New Drug Application (NDA) or Biologics License Application (BLA) for an adult cancer drug targeted at a molecular mechanism relevant to the growth or progression of a pediatric cancer must submit an iPSP. The Act applies to NDAs and BLAs for a new active ingredient, including biosimilars, filed on or after August 18, 2020, and applies even if the adult cancer does not occur in children or the adult indication was granted orphan designation.

The iPSP must provide an outline of the proposed molecular targeted pediatric cancer investigation “*using appropriate formulations, regarding dosing, safety, and preliminary efficacy to inform potential pediatric labeling.*” *(* 21). Furthermore, it should include any planned request for a deferral or waiver with supporting documentation. To design a pediatric trial, sponsors should utilize adult safety, pharmacokinetic (PK), and efficacy data and determine if an age-appropriate pediatric formulation is needed (22). The iPSP should address the following areas: Safety, Exposure, Dose/Exposure/Response (DER), Response, and Sample Size. Sponsors may want to refer to relevant guidance (21) and FDA’s lists of relevant and non-relevant pediatric molecular targets (23). FDA recommends that sponsors also consider including adolescents in Phase 2 trials and seek scientific advice from both FDA and European Medicines Agency (EMA) to avoid the need for duplicate pediatric studies. Additionally, requesting a consultation with the Oncology Center of Excellence Pediatric Oncology Program and the Oncology Subcommittee of the Pediatric Review Committee may be beneficial (24).

Recently the FDA launched an initiative to reform the dose optimization and dose selection paradigm in oncology drug development (14) and more recently issued draft guidance on ‘Optimizing the Dosage of Human Prescription Drugs and Biological Products for the Treatment of Oncologic Diseases’ (14) building on a previous whitepaper. The current focus is on targeted therapies with both small and large molecules while dose optimization for existing therapies and some modalities is not part of the current remit for Project Optimus. This results in excluding the evaluation of radiopharmaceuticals, cellular and gene therapy products, microbiota or cancer vaccines. While it is noted that guidance is lacking on how approaches to dosage optimization are applicable to pediatric oncology, there are steps are being made to address this (25).

## 3 Cancer types in pediatric population

Although rare, many types of cancer can affect children, ranging from common to rare. Four types of cancer (solid and non-solid carcinoma), which are commonly diagnosed in pediatric populations, are described below, extracted from **Table 1**.

1. Leukemia: This type of cancer affects the blood and bone marrow and is the most common cancer diagnosed in children. There are two main types of leukemia: acute lymphoblastic leukemia (ALL) and acute myeloid leukemia (AML), originating in the bone marrow. ALL is characterized by the growth and proliferation of immature lymphoblasts, leading to a decrease in the production of normal blood cells. AML is characterized by the abnormal proliferation of immature myeloid cells, disrupting the normal production of blood cells. These are the most common forms of childhood cancer, and ALL survival rates have improved from 10% to 90% with newly identified therapies through clinical trials with a focus on biomarkers and therapeutic strategies (7, 48–50).
2. Brain and Central Nervous System Tumors: These tumors can be benign or malignant and occur anywhere in the brain or spinal cord. There are more than 100+ types of brain-related tumors; most are exceedingly rare; e.g., Diffuse Intrinsic Pontine Glioma (DIPG) is an aggressive rare cancer with around 300 children in the U.S. each year (51). DIPG is typically diagnosed under 10 years of age and most prevalent between 6 to 9 years of age with a median survival of less than 1 year of age (52). This creates challenges such as patient recruitment and long-term safety follow-up in the clinical trial design. Neuroblastoma can be found at both birth and later in life. This creates an increased challenge to design a clinical trial with a board age spectrum.
3. Lymphomas: Lymphomas are cancers of the lymphatic system (a part of the immune system) which are common in children. The two main types of lymphoma are Hodgkin lymphoma (HL) and non-Hodgkin lymphoma (NHL). The presence of abnormal Reed-Sternberg cells characterizes HL and tends to spread in a predictable manner between lymph nodes. In comparison, NHL encompasses a broader range of lymphomas that involve several types of lymphocytes and can affect multiple lymph node groups and extra-nodal sites. HL is common in adolescents, while NHL is more common in younger children. Treatment and prognosis differ between the two, with HL generally having a higher cure rate and more standardized treatment approaches compared to the diverse subtypes and clinical behaviors of NHL.
4. Sarcomas: Sarcomas are cancers that start in bone or soft tissue, such as muscle or connective tissue. Examples of sarcomas that can affect children include osteosarcoma and rhabdomyosarcoma. Ewing Sarcoma is found to be more prevalent in Caucasians followed by Asians/Pacific and African Americans which may become a key consideration in the selection of sites for the clinical trial and the importance of collecting race-specific information for quantitative analysis (53).
5. It is important to note that many other types of cancer can occur in children, as shown in **Table 1**, and the prevalence of these cancers can vary depending on age, sex, and other genetic factors. **Table 1** also summarizes key considerations when designing clinical trials, including age, disease prevalence, current and potential new treatments and formulation, feasible PK measuring routes, and potential variables to be collected for safety, PD, and efficacy endpoints.

**Table 1:** Key consideration for some known childhood cancer when considering clinical trials.

Indication of childhood cancer	Typical Age of Incidence (year)	Prevalence	Existing and potential routes of administration	Current and potential treatments	PK relevant and sampling	Common safety endpoints	Biological effective and efficacy endpoints	Challenges and comparison to adult
Brain (26, 27), spinal cord tumors and Diffuse Intrinsic Pontine Glioma (DIPG) (28)	0 to <20y	~15-25% of pediatric cancer. Around 5.8 per 100, 000 population. Common.	Ultrasound disruption, intra-arterial, intravenous, intralesional, intrathecal, intraventricular, oral (29)	Surgery Traditional: Chemotherapy, radiation New: target mediated agents (30, 31): e.g., MAPK, BRAF, mTOR, MEK, HDAC, LSD1, PDGFRA, NTRK	Yes, blood PK, CSF sampling, direct tumor sampling with biopsy, PET with tracer, micro-dialysis	Depending on the MoA (32), nausea, vomiting, fatigue, headache, rash, myelosuppression, seizures, optical related AE, myelosuppression,	Main: Overall response rate (ORR), best overall response rate (BOR), duration of response (DOR) Response evaluated for solid tumor: RECIST/tumor dimension Others: kinase/t-cell biomarkers, blood cell counts, ctDNA, PFS, OS Intra-tumoral drug level concentration.	For PK in blood, longitudinal data collection is feasible while other types are very invasive, additional assay validation. is needed. Sample based on biopsy are limited. For efficacy, there are different response criteria for response rate and tumor size assessments. Longitudinal data is possible. It is less common change from low grade to high grade tumor in children. If condition is similar, children have better prognostic than adult. The survival rate is also higher in children.
Neuroblastoma	0-5 (mostly to children)	~6% and 90% of neuroblastoma are under 5 and rare above 10y	e.g., Intravenous, oral, parenteral, intraperitoneal, subcutaneous, intratumor, intrathecal, intracerebral, intradermal, intraarterial	Depending on the risk (low, medium, and high) groups due to how far the cancer has spread to bone marrow, bone, lymph nodes and abdomen. Surgery, muti-agent chemotherapy, myeloablative chemotherapy, radiation, total body irradiation, autologous stem cell transplant Targeted agents (33): Aurora A (34), ODC1, mTOR, pan-PI3K/mTOR, AKT, ALK, MEK, CDK, NTRK, HDAC, BCL-2, BRD4 inhibitor	Blood PK	Depending on the MoA (32), nausea, vomiting, fatigue, pain, rash, myelosuppression, GI toxicity	Main: ORR, DOR RECIST response, MIBG response, Bone marrow responses. Biomarker (blood or biopsy) based on International Neuroblastoma Response Criteria	Many targets (35) identified but many trials are ongoing to understand the MoA in both mono and combination investigation. Neuroblastoma in adult and pediatric achieve similar survival rate suggested in (36).
Leukemia →Acute Lymphocytic Leukemia (ALL) →Acute myelocytic leukemia (AML) Juvenile myelomonocytic leukemia (JMML) Acute promyelocytic leukemia (APL) Chronic Lymphoblastic Leukemia (CLL) →Chronic Myeloid Leukemia (CML)	0 to <18y, ALL peak around 2-5y, AML and JMML is more common in 2y. Leukemia (ALL, AML) in infants (<1y) is rare	~30% ALL (75% of total L), AML (20% of total L), rare for JMML, APL, CLL and CML	Oral and IV	Chemotherapy, radiation, stem cell transplant Azacitidine [Vidaza] -JMML Daunorubicin and cytarabine liposome [Vyxeos] – t-AML, AML-MRC, gemtuzumab ozogamicin [Mylotarg] - AML	Blood PK	myelosuppression, GI toxicity, hair loss, mucositis, fatigue, infection	Complete Remission (CR), Event-Free Survival (EFS), Overall Survival (OS), Minimal Residual Disease (MRD) negativity, Relapse-Free Survival Other biomarker such as white blood cell, CD (cluster of differentiation) markers	Heterogeneity of leukemia in adult and children (37).
Lymphoma: Hodgkin and non-Hodgkin (HL, NHL) Staging using PET scan	Rare in infant and not common, <3y, common in 10 to <18y	~7-11%	IV infusion, intravenous, oral, sub-cutaneous	Depending on the stages of the disease and types of HL or NHL using surgery (stage 1 and II) chemotherapy or combination with radiation. Other therapies such as Allogenic or autologous Stem Cell Transplant, bispecific antibody, CAR T-cell therapy, checkpoint inhibitor e.g., anti- PD-1 for HL	Blood PK	Depending on the MoA, nausea, vomiting, fatigue, pain, infection, myelosuppression,	Event free survival (EFS) and OS	Identify useful prognostic factor, more therapy for 1^st^ line and relapse (38).
Osteosarcoma (OGS)/bone cancer/Ewing Sarcoma (EWS)	10 to <18y	~1 to 5% Osteosarcoma rare in children <5y	IV infusion, intravenous	Surgery, chemotherapy (with methotrexate), and radiotherapy, denosumab [Xgeva] for giant cell tumor for bone	Blood PK	Myelosuppression,	Event free survival (EFS), OS, disease free survival, Quality of Life (QoL) Biomarker e.g., for osteosarcoma: Alkaline Phosphatase (ALP),	Rarity of the disease, lack of biomarker and targeted therapy. Long term safety to understand the impact to bone or growth development. Osteosarcoma in adult are mostly secondary, associated with prior therapy (39).
Hepatoblastoma (Liver Cancer)	Most common under 4y	~1%	IV infusion, intravenous	Surgery, and chemotherapy T-cell therapy ET140203 (ARTEMIS) (40)	Blood PK	Myelosuppression,	Event free survival (EFS), OS, Tumor response Serum alpha-fetoprotein (AFP) (41)	Rare orphan disease Difference between adult and children hepatoblastoma is still in research.
Retinoblastoma (eye cancer)	0 to 15y Common < 5y	~2-3%	IV infusion, intravenous	Surgery (enucleation), chemotherapy, cryotherapy, light coagulation, stem cell transplant, and radiation	Blood PK	Myelosuppression, ototoxicity	Event-free eye survival (EFES) and overall eye survival (OES) (42)	Few therapeutic options that with less risk of toxicity (43). Rare in adult.
Sarcoma (Soft tissue cancer): Rhabdomyosarcoma (Exclude OGS and EWS)	0 to <18y Peak in 1 to 5y	~5-8%	IV infusion, intravenous	surgery, radiation therapy, and chemotherapy Other targeted (44) therapy e.g., pembrolizumab, denosumab,	Blood PK	Myelosuppression,	OS and PFS	Standard PIP. Adult has lower survival than children with similar stage of disease (45).
Wilms Tumor (Kidney tumor)	Usually occur 3 to 4y	~5-6%	IV infusion, intravenous	dactinomycin [COSMEGEN]	Blood PK	Myelosuppression,	Disease-free survival (DFS) Tumor Shrinkage (TS), total Tumor Resection (TR), Event-Free Survival (EFS)	Adulthood Wilms tumor is rare.
Germ cell tumor	0 to < 18y	~2-4%	IV infusion, intravenous	Vinblastine sulfate	Blood PK	Myelosuppression,	Overall response (OR), PFS, OS	Rare as in childhood cancer but common in adolescent and young adult (46)
Melanoma	Rare in young and common in adolescent 18 to 18y	~1-4%	IV infusion, intravenous, oral	Surgery, chemotherapy, radiation and immunotherapy, ipilimumab, nivolumab, pembrolizumab	Blood PK	Myelosuppression,	OR, PFS, OS	Amelanotic and raised lesions), nodular histotype, and thick lesions are more common in children than adult (47).

### 3.1 Current pediatric oncology treatment modalities and new clinical trial challenges

The typical standard of care (SoC) treatments for pediatric oncology patients are surgery, CTx, and radiation therapy/radiotherapy (RTx). For some specific tumors, hematopoietic stem cell transplantation might be feasible (54, 55). It is the norm to give traditional SoC as part of combination therapies (56). However, challenges remain due to the toxicity caused by CTx/RTx or CTx+RTx, e.g., myelosuppression, which will result in dose reduction or drug holidays until the level of the concerned blood cell count returns within normal range. Other management approaches might be feasible to accelerate the resume of the patient’s cycle depending on the type of myelosuppression of concerns, e.g. in the case of neutropenia, co-medication such as the administration of G-CSF to increase neutrophil count; for anemia, iron supplement/red-blood-cell transfusion/recombinant human erythropoietin erythropoiesis-stimulating agents (ESAs) to stimulate the production of red blood cells, and for thrombocytopenia, platelet transfusion as a treatment and thrombopoietin agents as prevention for thrombocytopenia. The long-term impact (late effects) of CTx and RTx can include various organs e.g., the heart, lungs, brain, nerves, kidneys, thyroid glands or reproductive organs (57). Adults treated for cancer during childhood experience a high risk to their fertility, health risk for pregnancy complications, preterm labor, fetal, malposition and low birthrates (58, 59). Clinical trials can offer new opportunities for pediatric patients with newly diagnosed diseases and those with relapsed and refractory diseases by evaluating novel targeted agents (60) and other modalities such as immunotherapy (61) and CAR-T (62–64). Since most SoCs are effective, many developers seek combinations by adding targeted/novel therapies to the SoC. However, to improve treatment outcomes, reduce toxicity and drug resistance, there is an increase in the number of combinations of targeted agents with different pathways (65). Contemporary trial design can also help to evaluate the reproductive risk based on non-clinical toxicology studies and, where feasible, limits the cytotoxic dosage to decrease the risk to the reproductive potential of pediatric patients (58). There are many successful cases where a new therapy emerges that addresses an unmet need and can replace traditional SoC. However, additional new therapies are still warranted to extend the survival time and improve the quality of life (66).

In addition, there are challenges in moving targeted treatments into areas where SoC (CTx/RTx) are already effective, and data are needed to see the superiority of targeted agents over SoC in a head-to-head controlled trial. Also, the oncologists’ choice of therapies to use is dependent on the stage and progression of the disease and the prior line of treatments (67), which might slow down the adoption of newly approved agents.

## 4 Other challenges and opportunities

**Figure 1:**
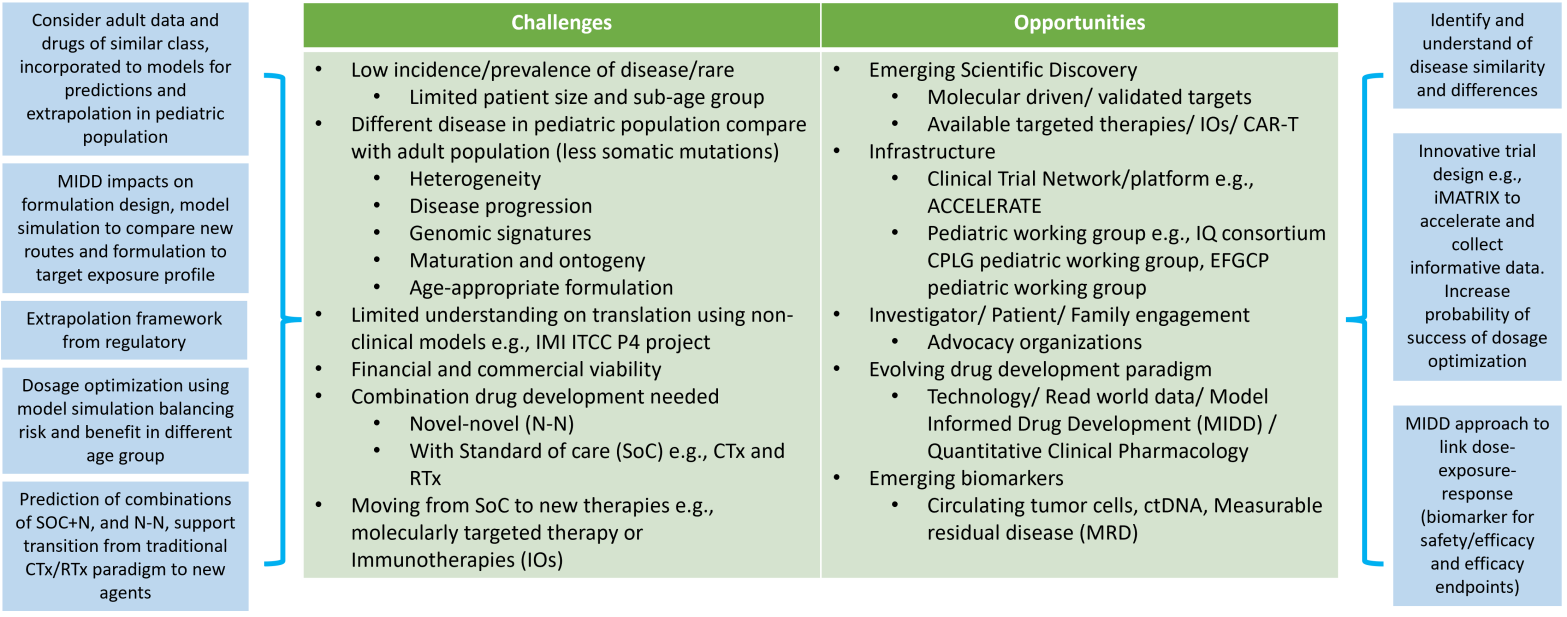
Challenge and opportunities when considering oncology pediatric drug development. Abbreviations: model informed drug development (MIDD), chemotherapies (CTx), radiotherapies (RTx), standard of care (SoC), chimeric antigen receptor T-cell (CAR-T) therapy, IMI ITCC-P4 (ITCC pediatric preclinical POC platform).

Working with pediatric oncology populations can be challenging due to several reasons outlined in **Figure 1**. The low incidence rates and disease prevalence result in small studies with long durations needed for recruitment. Cancer response to the treatment, in terms of disease progression, might highly vary between adults, children and children of different ages. The rapid and continuous physiological changes during growth and maturation mean that pediatric patients cannot be treated as ‘small adults’. These physiological differences include body composition (fat, bone, muscle, and water), height, body weight, organ size and functional maturation rate that impact how drugs are absorbed, distributed, metabolized, and eliminated in the body, as well as how the body responds to drug therapy from safety, tolerability and efficacy perspectives. Numerous publications provide key considerations to the impact of ontogeny on PK and PD (67–69) with a focus from birth to 2 years of age, where most of the functional maturation takes place and the rates of development of various parts are rapid during this time until reaching to adult levels. The bioavailability of orally administrated drugs impacts absorption and is dependent on multiple developmental factors such as the stomach pH level and gastric emptying time, e.g., pH values reach an adult level after 2 years of age (70, 71), intestinal drug-metabolizing enzymes (e.g., CYP3A4) and transporters (e.g., P-glycoprotein, P-gp). For drug that are subcutaneously or intra-muscularly administrated will depend on the fat and muscle compositions and dose adjustments might require for different age group in pediatric especially when less than 2 years of age. Distribution of the drug will depend on body water and fat ratio and various lipophilic and hydrophilic drugs. Plasma protein blinding tends to be low, especially in neonates, which will result in increased proportion of unbound drug (72). The level of enzyme expression in the liver will change the rate of hepatic metabolism. It is also important to understand the maturation to enable extrapolation from adult data into pediatric populations. Several literatures reviews (73–75) provide information for metabolizing enzyme ontogeny and how to incorporate it into physiological modelling (76). The glomerular filtration rate (GFR) is the key measure of renal function. GFR rapidly increases from birth and reaches adult value at 1 year of age (77). Getting actual GFR is challenging and complicated, especially in clinical trial settings. Estimated GFR (eGFR) or creatine clearance are often used to estimate kidney function. Several pediatric-specified equations have been developed for children, and comparisons of their strengths are still ongoing (78, 79). Drug response, adverse effects and efficacy effects in children can differ to adults due to maturation. For example, adverse effects that are related to the dose and the time on drug which are strongly related to the ontogeny in the ADME processes. Understanding the differences will allow practitioners to design optimal dose scheduling to incorporate sufficient levels of drug holidays to enable patients to recover or prevent of safety concerns. Modelling approaches can support the prediction of dose and schedule.

A way to increase the potential availability of oncology research and increase access to medicine to the pediatric population is to include the pediatric population in clinical trials early. In recent years, researchers (ACCELERATE platform (80, 81) and regulators with guidance to industry (82) strongly support the inclusion of adolescents above 12 years of age into adult phase 1 and 2 studies to increase access. This will benefit from collecting data in comparatively safer pediatric populations, as the significant maturation concerns lie within less than 2 years of age. This also provides benefits for adolescents with relapsed cancer, rare cancer or cancer which is not prevalent in < 12 years of age to have the opportunity to access potential treatment.

Development of an age-appropriate formulation is mandatory from a regulatory and clinical feasibility standpoint; new formulation forms, e.g., for oral administration route, young children would not be able to swallow tablets/capsules. Hence, a suspension/liquid would be needed. Other routes such as injectables, needle lengths, and sites (fat, venous, muscle) of administration also need to be investigated. The exposure from the new pediatric formulation is subjected to comparison to the efficacious dose in adults by comparing the targeted exposure without the need for a new study in pediatrics. Thus, the development of an age-appropriate formulation and dosage for pediatric is challenging (83).

The EMA reflection paper on formulation choice for the pediatric population (84) listed several formulations with various administration routes (e.g., oral, nasal, rectal, topical/transdermal, parenteral, pulmonary, and ocular) and appropriateness and preference of these formulations for various age. A lot of the decisions are also based on functional maturation. This information gives initial guidance to the research on the range of potential types of formulation to evaluate. For orally administrated drugs, taste (palatability), smell, color, dosage forms (tablet, capsule, liquid, powder), dosing flexibility, size/volume, texture, and mouth feel, are all part of the consideration and requirements for the design of age-appropriate formulation. Excipient selections are also crucial; some excipients might cause adverse effects in children and not in adult populations. Also, the level of preservative (83) used needs careful consideration to ensure safety in pediatric populations.

Invasive procedures should be minimized in children, especially when they are unwell. Therefore, there are limitations (due to ethical and feasibility reasons) to blood sampling collections for various age groups in terms of volume and frequency to gather necessary data (PK, clinical chemistry, blood count and blood-borne biomarkers) from clinical trials. Micro-sampling/small-volume capillary sampling is a good method and has gained popularity to minimize the blood sampling volume. However, careful design and planning on the blood sampling scheme should still employed, especially in the pediatric population with advanced cancer, with a risk of infection and clinician-induced anemia (85). Population PK modelling has been a good way to incorporate sparse PK for analysis to reduce the sampling requirement/design sparse sampling scheme and provide performance evaluation of micro-sampling techniques (86). Therefore, a quantitative framework is needed to utilize sparse pediatric samples or existing data from drugs in the same or similar class to better understand a new drug’s activity in children. Invasive procedures should be minimized in children, especially when they are unwell. Ultimately, unnecessary clinical trials should be avoided.

Lastly, sponsors must bridge the knowledge gap between adult and pediatric populations. This involves understanding the similarities and differences in disease progression, drug safety, drug effects, and the magnitude of the disease progression or dose-exposure-response (DER) relationships through non-clinical and clinical data. Often, real-world and published clinical data are used to form the basis of these extrapolation arguments but can also be limited, and therefore, stand-alone efficacy evaluations in pediatric cancer populations are often required to support approvals for pediatric indications.

## 5 Innovative trial design

### 5.1 Platform trial

To overcome the limited number of pediatric oncology patients due to the low prevalence and heterogeneity of the disease, master protocol study designs such as basket, platform and umbrella trials study designs can be considered. Although these trial designs are more often applied to adult populations to test new anti-cancer agents more effectively, regulatory agencies now highly encorage the application to the pediatric population in oncology and across diseases (87).

This type of trial design allows the testing of multiple therapies as mono or combination and several diseases in parallel under an overarching protocol instead of numerous individual protocols for every sub-study and conducted at different periods (88, 89). Basket trials (BT) aim to investigate one targeted therapy (alone or in combination) in various cancer types, while umbrella trials (UT) aim to evaluate multiple targeted therapies for one disease (a single type of cancer: can be with different mutations) to detect signal and confirm drug mechanisms.

BT and UT can be platform trials (PT), but the difference is that no new treatment can be included or excluded during the trial for BT and UT. Platform trial allows the investigation of multiple drugs and multiple diseases (tumors) populations to be added at different times; a common control group is also part of the setup to increase efficacy compared to individual controls. Examples of these types of trials include the NEPENTHE trial (90), Pediatric MATCH trial (91), iCat Study (92) and iMATRX trial (93). The iMATRIX platform studies multiple compounds across multiple ranges of relevant tumor types. For example, the iMATRIX-atezolizumab study (94) matched tumor biology with the mechanism of action to enroll patients in the trial and collect PK and safety data across various tumor types. Incorporation of innovative adaptive trial designs with a model-informed approach allows more efficient dose escalation in Phase 1 pediatric trials and, thus, more effective ways for dose-findings.

### 5.2 Adaptive and model-based design

Phase 1 pediatric oncology studies are typically small, open-label, sequential studies enrolling 3-6 patients per dose escalation (95). The starting dose in the pediatric trial is usually based on the MTD found in adults. Deriving a recommended Phase 2 dose, regimen, and potential combination option continues to be a goal through the escalation phase. Rule-based methods such as 3 + 3, rolling 6 (96), accelerated titration methods and pharmacologically guided dose paradigms used to identify the recommended dosage (95) based on clinical data generated during the pediatric oncology study. The disadvantages of these methods are that they cannot use all previous information on the study and cannot easily provide extrapolation to untested schedules. Model-based approaches for human studies (97) allow for utilizing all available data and determining the relationship between dose, exposure, and effect. The potential and application of various escalation approaches such as the Time-to-event Continual Reassessment Method (TiTE CRM), escalation with overdose control design (EWOC) and combined TiTE-EWOC are shown in (98), where EWOC design was used in a pediatric trial for irinotecan and gefitinib combination (99), and for crizotinib (100) and incorporating the examples to illustrate the practical implementation of model-based approaches (e.g., EWOC used in irinotecan and gefitinib) and the challenges they address.

The first-time-in-human trial described (101) provides an example of incorporating model-based approaches, which can inform the pediatric dose escalation approach. This approach aims to include prior information, e.g., non-clinical and adult data, which was reviewed to identify and prioritize key data for analysis that would provide useful signals for tolerability. The predicted PK profile was used as prior information to enable analysis of the sparse datasets emerging from the first few cohorts. Pharmacodynamic and safety models developed from prior data were re-applied to the emerging clinical data. The models were updated with data from each successive cohort of patients and then used to simulate the endpoints for a range of proposed dose escalations to inform the clinical team of predicted outcomes.

These models were also used to explore options for further arms of the study to investigate alternative schedules. Even from small data sets, the models developed were robust to inform escalation. This was partially demonstrated by the ability to predict untested doses and schedules. The simulations of continuous variables allowed for dose increments and the starting dose for alternative schedules to be determined quantitatively. This included the instigation and escalation of intermittent dosing arms that proceeded to identify the recommended dose more quickly than would have been the case with a classical approach.

The utilization of non-clinical, clinical PK, safety and PD data in model-based dose escalation allows rapid learning in early-phase clinical development. This real-time approach allows simulation of scenarios based on the available information enabled the development program to identify the recommended dosage for a range of schedules efficiently, thereby improving trial outcomes and implementing a missing dose management strategy. Management of missing doses is important especially for pediatric trials to ensure all the data collected are optimal (101). This approach also provided a simulation profile to guide clinical investigators to maintain the optimal dosage by showing the wash-out period and when the planned optimal dose and schedule could be resumed.

## 6 Roles of MIDD: focus on dose finding, justification and extrapolation

MIDD approaches (102) have been widely embraced by regulatory agencies worldwide, including the US Food and Drug Administration (FDA), the European Medicines Agency (EMA), and the Pharmaceuticals and Medical Devices Agency of Japan. MIDD methods can play a significant role in achieving the goal of improving pediatric oncology therapies. MIDD helps to increase understanding of the relationship of various biomarkers with efficacy endpoints and pharmacokinetic parameters. In addition, data from all age groups can be used to inform models and simulations for younger groups. However, caution is needed as drug responses depend on the stage of disease progression and mechanism of action of the drug. Regulators and the pharmaceutical industry strongly endorse the MIDD approach to support pediatric drug development. The International Council for Harmonisation (ICH) E11(R1) addendums also provide guidance for applying modeling and simulation to pediatric drug development. The early pediatric strategy should include multidisciplinary experts in the use of modeling, available data, and the assumed clinical setting.

Identifying the starting dosage in pediatric oncology trials is still challenging, especially for therapies intended only for a pediatric population, despite the availability of various quantitative approaches. For example, the recommended Phase 2 dose (RP2D) and MTD are often several-fold higher than the starting dose, which could be due to the uncertainty of the drug exposures and the safety of the starting dose. The method most commonly used for setting the starting dose is based on an empirical dose scaling approach rather than utilizing all the information by applying a population pharmacokinetic (PK) or physiologically based pharmacokinetic (PBPK) modeling approach if exposure matching is deemed to be feasible.

Population PK studies in pediatric populations involve collecting PK data from children of various ages and sizes, typically in a clinical setting. The data are then analyzed using modeling and simulation techniques to characterize the typical PK parameters and their variability within the population. PK models incorporating allometric scaling based on body size metrics can facilitate the inclusion of adult data to determine the optimal starting dose and schedule for the initial pediatric trial.

When scaling adult doses to a younger pediatric population (e.g., < 2 years of age and especially with neonates), maturation and physiology need to be considered with a PBPK approach, which allows these differences to be incorporated into the predictions. In a typical PBPK workflow, the starting point is normally a model validated based on adult PK data, with associated system parameters, and physicochemical drug information. Then the physiological parameters are adjusted to reflect the pediatric age range and ontogeny being considered, which allows pediatric drug exposures to be predicted and a potentially effective starting dose to be selected. An illustration of the PBPK workflow is shown in the lisinopril example (103). For older age groups, a weight-based scaling approach using population PK may be appropriate if weight is found to be a significant covariate.

Additionally, PBPK models that account for ontogeny, physiological changes, and disease progression can simulate drug profile and drug-drug interactions, predict responses for various age groups, and extrapolate data from adult to pediatric populations.

There are several examples in the literature where PBPK approaches have been used to assist pediatric oncology drug development, e.g., olaparib using the PBPK approach to setting doses in the pediatric population is shown in **Table 2**. This approach ranges from selecting a starting dose in a first-in-pediatric trial to evaluating the factors that might influence drug exposure in children and optimizing trial design; for example, setting the PK sampling schedule and windows. Other semi-mechanistic models, such as the myelosuppression model, can be applied to characterize the dynamics of various blood cell types, such as absolute neutrophils (108) and platelet counts. These models can predict the time course and recovery of cell counts during drug treatment and how these translate to the occurrence of adverse events (AEs). They can also be further adapted to evaluate multi-cell types to support dose/regimen findings. In addition, model-based prediction (109) can also recommend that increasing the frequency (e.g., daily instead of the typical limited clinical monitoring) of the absolute neutrophil counts during chemotherapy myelosuppression can improve therapy management.

**Table 2:** Modeling example using quantitative approach to impact on dosage selections.

Modeling example	Approach	Details	Impact
Naxitamab-gqgk (DANYELZA) (104)-combination with GM-CSF for pediatric less than 1 year of age with relapsed or refractory high risk neuroblastoma in bone/bone marrow	PopPK, PKPD to dose adjustment and optimize risk benefit profile	The FDA assessment revealed that the sponsor has clinical data for naxitamab IV infusion at a dosage of 3 mg/kg/day on days 1, 3, and 5 of each 28-day cycle (totaling 9 mg/cycle). Population pharmacokinetic analysis showed that naxitamab exposure increases with higher body weight, particularly in patients weighing 70 kg to 80 kg compared to those weighing less than 50 kg. However, limited data exists for patients weighing over 50 kg receiving doses above 450 mg per cycle. To address this, the FDA recommends a dosing cap of 450 mg per cycle for patients with a body weight over 50 kg. By implementing this cap, naxitamab exposures in patients with a higher body weight are comparable to those with lower body weights (30 kg to 50 kg) using a weight-based dosing of 3 mg/kg/day (9 mg/kg per cycle).	Rare disease and no additional clinical trial for dose justification needed for body weight over 50 kg. Approved for pediatrics aged 1 year and older, and adult patients with relapsed or refractory high-risk neuroblastoma in bone/bone marrow.
Pembrolizumab (KEYTRUDA) in children with a PD-L1 positive advanced, relapsed or refractory solid tumor or lymphoma	Robust model developed in adult and updated with emerging pediatric data from pediatric study	According to the EMA assessment, the sponsor utilized population pharmacokinetic (PopPK) analysis to extrapolate the effectiveness of pembrolizumab in adult patients with relapsed or refractory classical Hodgkin lymphoma (rrcHL) to pediatric patients with rrcHL. This extrapolation was done through a model-based PK bridging analysis, which involved comparing the pharmacokinetics (PK) and exposure levels of pembrolizumab in various age groups of pediatric patients to the data obtained from studies conducted on the adult population. Besides the pharmacokinetic (PK) analysis, the pediatric study also offered additional information regarding the effectiveness and safety of pembrolizumab in pediatric patients diagnosed with relapsed or refractory classical Hodgkin lymphoma (rrcHL).	The therapeutic indication for KEYTRUDA is being expanded to include an earlier line of therapy and the inclusion of pediatric patients. KEYTRUDA, as a standalone treatment, is now indicated for both adult and pediatric patients aged 3 years and older who have relapsed or refractory classical Hodgkin lymphoma and have experienced treatment failure with autologous stem cell transplant (ASCT) or have undergone at least two prior therapies when ASCT is not a viable treatment option.
MEK Inhibitor, trametinib in children and adolescents subjects with cancer or plexiform neurofibromas and trametinib in combination with dabrafenib in children and adolescents with cancers harboring V600 mutations	PopPK	The four-part design enabled efficient dose finding for both trametinib monotherapy and combination therapy guided by PK analysis to model similar exposures established in adults treated at the approved doses (105). Incorporation of PK as a driver of dose decisions enabled patients to reach therapeutic levels of exposure without dosing to excess or finding a maximum tolerable dose. Based on the PK and DLTs, the recommended age-based and weight-based pediatric dosing of trametinib was established to be 0.032 mg/kg once daily for patients age < 6 years and 0.025 mg/kg once daily for patients age ≥ 6 years.	There were no adjustments made to the RP2Ds in pediatric doses for combination therapy compared to the established doses for each monotherapy. The pediatric dosing for dabrafenib monotherapy had been previously determined and was found to be well tolerated with no noticeable evidence of drug-drug interactions.
Dasatinib (106) approved in adults and pediatrics for treating Philadelphia chromosome-positive (Ph+) chronic myeloid leukemia in chronic phase (CML-CP) and Ph+ acute lymphoblastic leukemia (ALL)	PopPK and PKPD	The pediatric development program for dasatinib relied on extrapolation principles that considered the similarities in disease and treatment response between adult and pediatric patients of various age groups, specifically focusing on Philadelphia chromosome positive (Ph+) CML-CP and ALL. This approach allows for the extrapolation of efficacy data from adults to the proposed indication without the need for a fully powered confirmatory Phase 3 trial in pediatric patients. During the Phase 1 study, no maximum-tolerated dose (MTD) was identified when dosing was based on body surface area at 60, 80, 100, and 120 mg/m^2^ once daily. Comparable dasatinib exposure was observed in pediatric subjects receiving 60 mg/m2 compared to adults receiving 100 mg, which served as the target exposure for weight-tier dose recommendations and the development of a formulation suitable for pediatric patients of different ages. To gain insights into the absorption mechanism and the impact of various factors such as physicochemical properties, absorption characteristics, and inherent differences in dosage form transit behavior on dasatinib bioequivalence, an integrated PB/PK model was developed.	Simulations were employed using the PB/PK model to extrapolate the pharmacokinetic (PK) data in pediatric patients with ALL, given the lack of observed PK data. These simulations aimed to extend the understanding of PK derived from monotherapy in CML patients to the combination of dasatinib-chemo in ALL patients. Additionally, the simulations served to provide additional support for the dosing recommendations based on body weight for the new formulation.
Olaparib dosing recommendations: bridging formulations, drug interactions, and patient populations (107)		PBPK-based model experiments were conducted to investigate the potential drug-drug interactions (DDIs) involving olaparib, specifically when it was administered as a victim alongside CYP3A inhibitors and CYP3A inducers. The experiments also explored olaparib as a perpetrator when co-administered with CYP3A, P-gp, or UGTA1A probe substrates. Furthermore, the models were employed to assess the pharmacokinetics of olaparib in different scenarios. This included examining the effects of mild, moderate, or severe renal or hepatic impairment on the pharmacokinetics of olaparib (both tablet and capsule formulations). Additionally, the models were utilized to determine the pharmacokinetics of olaparib tablet formulation in pediatric subjects.	The model was utilized to simulate the exposure of olaparib in scenarios where there is a lack of clinical data, particularly in populations with severe renal or hepatic impairment. These simulations helped generate initial dosing recommendations for olaparib in pediatric patients. By using the model, researchers were able to estimate the expected exposure levels and adjust the dosing recommendations accordingly to ensure safe and effective use of olaparib in these specific populations.

MIDD approaches can also support dosage optimization, provide evidence of efficacy, enhance clinical trial design, and reduce or eliminate the need for clinical trials under certain conditions. Some approaches are shown in **Table 3**. Examples of where MIDD has been used include dasatinib for Ph+, CML-CP and Ph+ALL, naxitamab-gqgk in combination with GM-CSF for high-risk neuroblastoma in bone and bone marrow, pembrolizumab in a PD-L1+ advanced relapsed or refractory solid tumor or lymphoma and trametinib and trametinib + dabrafenib in cancer with V600 mutations. These quantitative MIDD methods allow sponsors to utilize the available data and effectively prepare the necessary components for their iPSP and PIP. Further research is needed to fully realize the potential of MIDD in advancing pediatric oncology drug development.

**Table 3:** Types of useful modeling approaches and notable applications for pediatric drug development and potential opportunities for dosage optimization.

Modelling approaches (67)	Why is it useful in pediatric drug development?	What’s the opportunity for oncology dose optimization?
Empirical and semi-mechanistic popPK	Allows description of the PK profile. PK models (110–113) can provide valuable insights into dose selection and optimization in pediatrics. They help determine appropriate dosing regimens, estimate drug exposure, predict drug concentrations, and evaluate the impact of covariates on drug disposition and variability between patients and occasions. By integrating population PK models with pharmacodynamic (PD) data, researchers can also explore the relationship between drug exposure and clinical response.	Essential of part of dose optimization to provide the PK model for computation for exposure metrices. The exposure metrics can then use for ER analysis. PopPK model Bayes estimates can use for PKPD model.
PBPK	PBPK modeling (114–116) allows factors such as body weight, body surface area, age, organ maturation, and developmental changes in drug-metabolizing enzymes and transporters to be incorporated into a model. This is especially useful for young populations (neonates and infants).	Very useful to support younger age group dose optimization especially on neonates and infants. Very useful for dose justification due to DDI, food effect and formulation effects which is not commonly investigated in pediatric population.
PK-safety/ER modeling/Concentration-ECG analysis	Understand the exposure safety or PK-safety relationship is important for dose findings during escalation phase and justification for final dose. Age might be an impact to CTC AE grade (117) e.g., children might have higher prevalence for fatigue than adult. For longitudinal marker, such as renal functions and blood cell count (118, 119) will also impact by age, and specific formular to derive renal function will be needed.	Important components to form the therapeutic index to guide dose findings.
PK-efficacy/ER modeling/time to event analysis	Understand the exposure PD/efficacy or PK-PD/efficacy relationship is important for dose findings during escalation phase and justification for final dose. Surrogate biomarkers, response rate (120) and tumor size modeling.	Important components to form the therapeutic index to guide dose findings.
Quantitative System Pharmacology (QSP)	QSP models (121) integrate existing mechanistic knowledge on disease (pathway information) and drug PK/PD in a quantitative framework allowing simulation of virtual trials, where candidate drug combinations are evaluated *in silico* before being tested in the clinic.	QSP model available for Immuno-Oncology platform which allow extrapolation from population to population including patient and pediatrics. An example and review are found by Chelliah et al. (122).
**Notable Applications**	**Why is it useful in pediatric drug development?**	**What’s the opportunity for oncology dose optimization?**
Simulation	Simulation based on developed model allow prediction of new dose and regimen required. E.g., Clinical Trial Simulation.	Simulation can show the prediction confidence and variability in the population and whether there is a clear distinction in term of safety and efficacy for dose selection.
Extrapolation	If the reference populations (e.g., adult or adolescent) are deemed to have similar disease to the target population, then efficacy is feasible using exposure matching (123).	If there are sufficient data (including dose ranges), extrapolation is an efficient way to omit an unnecessary trial and set the dose in a particular population.

The IQ Clinical Pharmacology Leadership Group Pediatric Working Group published a white paper explaining extrapolation’s role in pediatric development (124). There are examples of different therapeutic areas, including oncology. The paper found that safety data in children is still required in many cases because of the concerns for potential toxicity of anti-cancer treatments. However, extrapolating the drug exposures can still be especially useful to support starting dose selection and escalation. This paper also gave information on applying the MID3 (a predecessor to MIDD) strategy to support a pediatric extrapolation plan. When developing a new targeted treatment for pediatric cancer, there are uncertainties in the relevance of the drug’s mechanism and the required drug exposures. Non-clinical cancer models have supported drug discovery and development for many years in the adult setting. The Innovative Therapies for Children with Cancer Pediatric Preclinical Proof-of-concept Platform (IMI ITCC P4) (124) aims to develop and characterize animal models for pediatric cancer that will ultimately support pediatric cancer drug development by using the data obtained from different childhood tumors in mouse models for translational pharmacology. It will also guide us on when the pediatric plan should be started in drug development.

Overall, MIDD/modeling and simulation studies in pediatrics are crucial in improving drug therapy in children by providing evidence-based dosing guidelines, optimizing drug efficacy, and minimizing the risk of adverse effects. Increased understanding of tumor biology, especially molecular drivers, could lead to immunotherapy being used as precision medicine. It would enable us to understand disease progression and differences in diseases in subpopulations such as pediatrics. The availability of data on emerging biomarkers also encourages using a model-informed approach to relate dose-exposure, dose-response, and ultimately dose-exposure, response relationships. Finally, using model-informed approaches can support strategies for drug combination regimens, either combining novel treatments together or with the standard of care.

## 7 Real world data

Real-world data (RWD) plays a key role in pediatric drug development (125, 126) and oncology (127), as it can help to overcome the challenges of collecting sufficient pediatric data from clinical trials. RWD approaches can be used throughout the drug development process. The use of RWD can provide insights into the safety and efficacy of drugs in pediatric populations and can support the development of more effective therapies for children with cancer. While in the context of pediatric oncology, RWD has predominately been limited to expanded access programs that provide supportive evidence for regulatory approvals (127, 128), there is potential to utilize these types of data in other ways.

In the field of pediatric oncology, RWD can be particularly valuable due to the rarity of the disease and the difficulty of conducting large-scale clinical trials. Initially, RWD can help identifying patient populations and clinical needs (129). This information can be used to prioritize drug development efforts and to design clinical trials that are more likely to be successful. For example, RWD can be used to identify children with rare or aggressive forms of cancer who are not responding to current treatments. RWD can be used to evaluate but also monitor the safety and efficacy of drugs in real-world clinical practice and to identify any adverse events that may not have been captured in clinical trials. This information can be used to inform future clinical trials and to improve the design and implementation of clinical studies. Furthermore, RWD can be used to bridge knowledge gaps between adult and pediatric patient populations and to better understand the extent of disease similarity and progression in different age groups. In addition, RWD can be used to validate MIDD (130) models; this can be done by comparing the predictions of the model to the observed outcomes in real-world settings and thereby increasing the confidence in the predictions made. This information can be used to inform the development of more targeted therapies and to improve patient outcomes. Overall, the use of RWD in pediatric drug development and oncology has the potential to provide valuable insights into the safety and efficacy of drugs in children, to support the development of more effective therapies, and to improve patient outcomes. However, it is important to ensure that RWD is collected and analyzed in a rigorous and standardized manner to ensure the validity and reliability of the findings (131).

## 8 Discussion and future

Cancer in children is rarer than in adults yet presents a high unmet need due to the limited number of treatment options, the heterogeneity of cancer, and the aggressiveness of the disease in children. Pediatric studies cannot be waived for a drug program based on the class of treatment if the mechanism is thought to be relevant in pediatric cancer. Thus, it is essential to evaluate the drug mechanism in pediatric oncology is relevant as early as possible and whether similar drug exposure as adult cancer patients is required for efficacy in pediatrics. Therefore, drug development must be efficient to deliver a new treatment to pediatric patients or by evaluating as early as possible to stop the development if the treatment does not show promise for pediatrics. Efficient trial designs must be coupled with quantitative approaches such as MIDD to minimize the number of pediatric patients required and the time needed to answer important clinical questions in order to accelerate much-needed cancer treatments for our youngest patients.

Pediatric oncology patients require safer and more effective therapies. The Research to Accelerate Cures and Equity (RACE) for Children Act is expected to play a crucial role in making this goal a reality. By effectively implementing MIDD, it can support optimal dosing, identify potential risks and benefits of drug products under development, enhance clinical trial efficiency, reduce the burden of trial participation, and eventually improve patient recruitment and retention, thereby increasing the likelihood of regulatory approval. The successful implementation of MIDD has the potential to revolutionize pediatric oncology drug development and improve outcomes for children suffering from cancer. Further research and development efforts are needed to fully realize the potential of MIDD in advancing pediatric oncology therapeutics. In addition, harmonization across different regulatory bodies is still warranted, and the oncology communities are actively encouraging the regulatory agencies to increase the number of targeted therapies that received a pediatric label indication (16, 132–135). Despite these challenges, dosage optimization is an essential part of developing new cancer drugs for children and adults. By carefully considering the unique factors that affect children’s response to drugs, researchers can develop safe, effective, and tolerable treatments for young patients.

## Author contributions

SYAC and JH designed the scope of the manuscript. JH and SYAC wrote the first draft of the manuscript. JH, SYAC, Y-WL, JB and RD wrote sections of the manuscript. All authors contributed to the manuscript revision and read and approved the submitted version.
